# Controversies in contrast-enhanced ultrasound (CEUS): pregnancy, paediatric, abdominal trauma, complex renal cysts, and endovascular aortic repair follow-up

**DOI:** 10.1186/s13244-025-02055-w

**Published:** 2025-08-15

**Authors:** Paul S. Sidhu, Dirk Andre Clevert, Annamaria Deganello, Maciej Piskunowicz, Vito Cantisani, Thomas Fischer, European Society of Radiology (ESR)

**Affiliations:** 1 Department of Imaging Sciences, School of Biomedical Engineering and Imaging Sciences, Faculty of Life Sciences and Medicine, King’s College London, London, UK; 2 Department of Radiology, King’s College Hospital NHS Foundation Trust, London, UK; 3 Interdisciplinary Ultrasound-Center, Department of Radiology, University of Munich, Grosshadern Campus, Munich, Germany; 4 Radiology Department, Medical University of Gdansk, Gdansk, Poland; 5 Department of Radiology, Policlinico Umberto I, Sapienza University of Rome, Rome, Italy; 6 Department of Radiology, Interdisciplinary Ultrasound Center, Campus Charité Mitte, Charité, Universitätsmedizin Berlin, Berlin, Germany; 7 Freie Universität Berlin, Berling, Germany; 8 Humboldt-Universität zu Berlin, Berlin, Germany; 9 Berlin Institute of Health, Berlin, Germany; 10 European Society of Radiology, Vienna, Austria

**Keywords:** Trauma, Pregnancy, Paediatric, Ultrasound, Contrast-enhanced

## Abstract

**Abstract:**

The use of contrast-enhanced ultrasound (CEUS) in clinical practice is theoretically limited to the licensed indications: focal liver lesions, breast, peripheral arterial system, and the heart. In reality, there has been a continuous expansion of the deployment of CEUS examinations to many other organs and body parts over the last 20 years. Many of these applications are a natural extension of the diagnostic capabilities of the CEUS examination, used to achieve a better imaging outcome. These applications have been supported by guidelines issued by scientific societies, detailing the application, accuracy, and safety of the clinical performance. Nevertheless, there are some areas in which it remains more difficult to establish the use of CEUS in the diagnostic pathway. In the pregnant patient, CEUS is an ideal examination—a natural extension of B-mode ultrasound, avoiding ionising radiation and iodinated contrast. The contrast agents used in ultrasound do not cross the placental barrier. Ultrasound in the paediatric patient is used widely, and extending this to a CEUS examination improves diagnostic capabilities, avoiding less child-friendly imaging techniques. The parent can be in the room at the time of the ultrasound examination. Other aspects of CEUS usage are hampered by the lack of physician engagement despite the proven advantages of the technique, the reduction in the morbidity associated with CT and MR imaging, particularly the contrast agents used in these modalities. Complex renal cyst classification, follow-up of blunt abdominal trauma and the surveillance following placement of an aortic stent graft are all areas of potential benefit to the diagnosis. All these are better imaged on a CEUS examination. Furthermore, cost savings can be achieved using CEUS, mostly by alleviating downstream costs of CT and MR imaging.

**Critical relevance statement:**

CEUS use outside licensed uses is becoming established, driven by the unique ability to achieve diagnostic standards safely and with patient acceptability, pushing the boundaries in areas of abdominal trauma, pregnancy, paediatrics, aortic implants, and complex renal cysts.

**Key Points:**

- CEUS has a narrow range of licensed applications in medical imaging, but is used widely.
- An exclusively intravascular agent allows assessment of vascular flow at the capillary level.
- CEUS is extremely safe and can be used in many areas that require repeated high-resolution imaging.

## Introduction

The introduction of contrast agents into ultrasound (US) practice has been a slow process despite the enthusiastic views of the proponents of this technique; adding a contrast agent to a basic US examination is not undertaken as often, with the natural default of the radiologist to acquire further cross-sectional imaging if there is uncertainty. Although this is often the view of the examining radiologist, this is not the privilege of the non-radiologist physician, whose access to further cross-sectional imaging is a more arduous pathway. More non-radiologist US practitioners have added a contrast-enhanced ultrasound (CEUS) examination to their toolbox, using this technique to good effect, with ultimate patient benefits. Part of the hesitation on progressing to a CEUS examination is the licensing of the US contrast agents, restricted to use in assessing focal liver lesions, peripheral vascular system, breast tissue and the heart. Aside from cardiology, where the ‘microbubble’ cardiac investigation is commonplace and indispensable, the use of CEUS in breast US is restricted (a biopsy is definitive and low risk), and peripheral vascular disease has diminished requirements for enhancement (Doppler techniques are sensitive).

In practical terms, the only radiological aspect that US contrast agents are ‘licensed’ for is focal liver lesions, a very narrow practical application. Notwithstanding these restrictions, an enormous number of innovative applications outside the liver have emerged, often a fortuitous discovery, and often a logical extension to the basic US examination. This raises medicolegal issues, which have been addressed in the past [1] and with the European Federation of Societies in Medicine and Biology (EFSUMB) issuing guidelines on non-hepatic applications [2, 3] and endorsing statements on non-licensed applications [4, 5]. The curious notion that a truly intravascular contrast agent, that is safe [6, 7], and is present throughout the vascular system after injection, can only be assessed through a narrow set of organs lacks credibility. Indeed, a ‘whole body’ approval has been suggested for US contrast agents in the United States [8], where reimbursement is a strong driving force for the development of CEUS applications. There is no organ limitation with contrast agents used in computed tomography (CT) and magnetic resonance (MR) imaging.

A review of the most controversial areas of the application of CEUS in non-licensed areas of practice will be discussed, and evidence presented as to the legitimate application in the interests of the patient.

## Pregnancy

One of the most frequently used imaging modalities in obstetrics is US; B-mode US has an excellent safety profile and is widely available. Even though the application of US in pregnancy may lead to potential harmful effects by local tissue heating, no foetal or maternal adverse effects of its use during pregnancy have been reported to date [9, 10]. However, there are a variety of clinical situations in pregnant patients where more elaborate imaging techniques and the requirement for a contrast agent are necessary. This is often performed with CT or MR imaging [11, 12]. Up to now, there is no official approval of CEUS during pregnancy, e.g. by the EFSUMB or by the World Federation for Ultrasound in Medicine and Biology (WFUMB) or the International Society of Ultrasound in Obstetrics and Gynaecology (ISUOG) [1, 13, 14]. This is despite evidence that ultrasound contrast agents used in medical practice do not cross the placental barrier [15–17].

### Advantages

With no clear evidence for the safety of CEUS during pregnancy, MRI is utilised when further imaging evaluation is necessary, and then application of gadolinium-based contrast agents is an option, which is controversial during pregnancy [10, 18]. The American College of Radiology (ACR) emphasises the avoidance of intravenous application of gadolinium agents in pregnant patients, indicating that it should only be used if absolutely necessary [19, 20]. Life-threatening emergencies during pregnancy require imaging with CT and the benefits of establishing the diagnosis, which outweigh potential foetal risks, including carcinogenic and teratogenic effects, due to ionising radiation [10, 21].

### Evidence summary

**Fig. 1:**
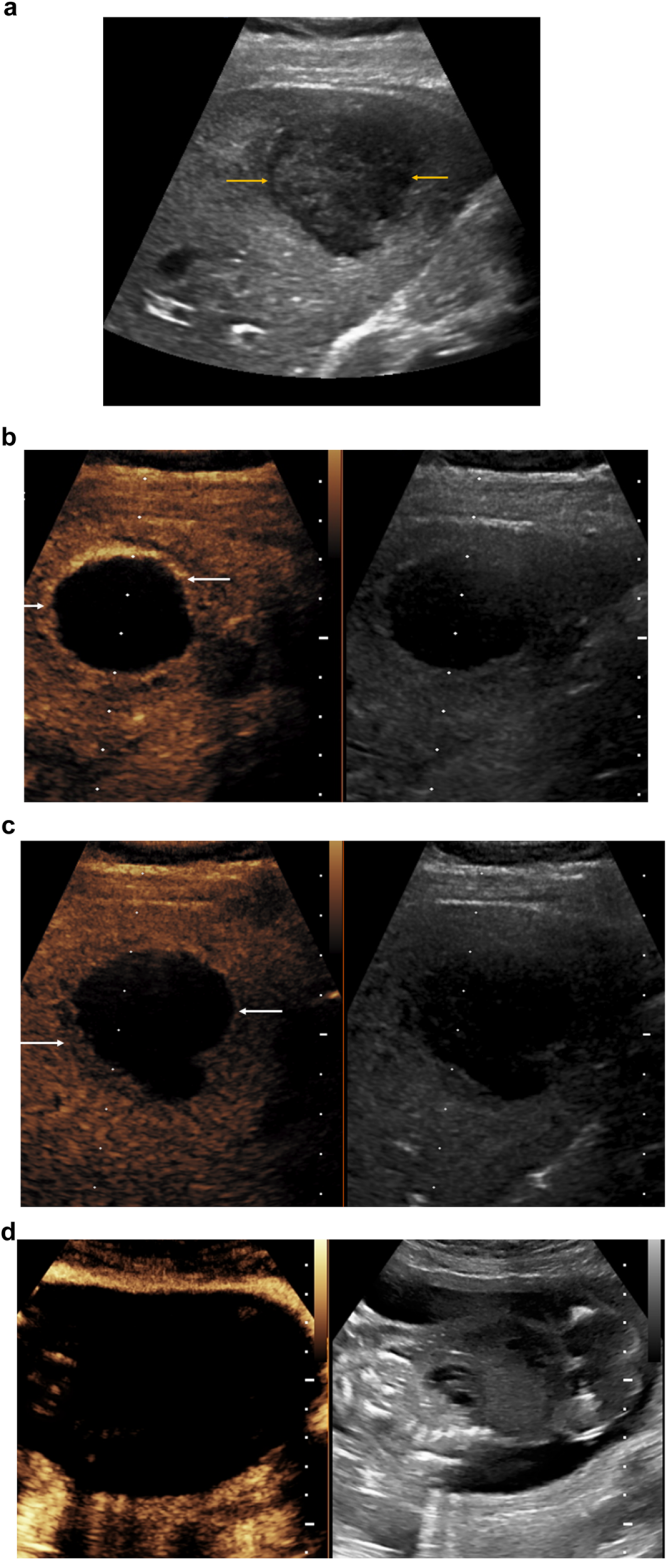
A 29-year-old woman in the eighth week of pregnancy presented with upper abdominal pain. An unenhanced MR examination detected a focal liver lesion, but could not offer a firm diagnosis, with a differential diagnosis of possible necrotic tumour, pyogenic abscess or a hydatid cyst. **a** The baseline B-mode ultrasound demonstrates the low reflective focal liver lesion (arrows) in the left liver lobe, with no distinguishing features. **b** Following the administration of 2.4 mL of SonoVue™ (Bracco SpA, Milan) there is no enhancement of the central aspect of the lesion, with peripheral rim enhancement (arrows) typical of an abscess. **c** On the later phase of the CEUS examination, washout of the rim was noted (arrows). A directed aspiration of the cystic structure was performed with the final diagnosis of amoebic abscess. **d** The extension of the ultrasound examination to the pregnancy sac, demonstrates no contrast enhancement of the foetus

Published data on CEUS during human pregnancy is sparse, mainly detailing the safe use of CEUS for assessing uteroplacental blood flow, caesarean scar pregnancy and invasive placenta percreta [22–24]. However, the future availability of data concerning safe off-label use of CEUS during pregnancy is likely to remain limited. One proposed prospective study regarding comparing CEUS and colour Doppler US in the first trimester assessment of the vascularisation of the placenta has not reported findings [25]. Case reports and case series studies regarding the utility of CEUS in pregnant women have demonstrated promising results, with one case report focusing on the use of CEUS for diagnosing liver echinococcosis in a pregnant patient [26], another confirmed the safe use of CEUS for evaluating hepatic lesions in six pregnant patients [27] and another reporting several acute abdominal scenarios requiring imaging in five patients [10] (Fig. 1).

Nevertheless, the application of CEUS during pregnancy has not been formally approved by leading societies for obstetrics and US due to concerns about potentially related adverse effects [13]. One of the benefits of CEUS is that it is a non-ionising imaging modality, as opposed to CT, that can be safely deployed in patients with hyperthyroidism, renal failure or allergic reactions to iodinated contrast media. Using gadolinium as a contrast agent with MR imaging raises the concern for long-term gadolinium retention, currently unknown in the foetus [20, 28], and concerns of gadolinium contamination of the environment [29]. Ultrasound contrast agents can be used to examine the micro-perfusion of parenchymal lesions of unknown entity or inflammatory foci, theoretically avoiding any hazards of long-term consequences of the agent crossing the placental barrier, as the microbubble contrast agents are truly intravascular and do not cross into the foetal circulation [30, 31]. Due to the limited amount of CEUS data in obstetrics, more research is needed; however, this technique in pregnancy is undoubtedly an important addition to the armamentarium.

### Future perspectives

With the lack of licensing in pregnancy, the application of CEUS in pregnancy will, in the near future, be limited to a ‘case-by-case basis’. With the accumulation of evidence on the basis of multiple cases, safety evidence will be obtained, and more confident application of CEUS in pregnancy will ensue.

## Paediatric practice

CEUS has emerged as an ideal diagnostic tool in children, due to its lack of radiation exposure, non-iodine content, and potential for widespread availability in many clinical scenarios. However, even though use has increased over the last few years in many different applications, intravenous use of CEUS in children still elicits controversy centred on safety, efficacy, regulatory barriers, and ethics [4, 32, 33].

### Advantages

One of the main areas for debate regarding the use of CEUS in children focuses on safety. While there is copious evidence in the literature supporting its safety in adults with minimal side effects [7, 34–37], there is still limited paediatric data [32, 38, 39]. Critics of US contrast agents argue that adult data may not be transferable to children and neonates, given their distinct physiological characteristics; in particular, neonates have immature immune systems, which may make them more vulnerable to possible side effects. In this regard, premature babies may have an increased theoretical risk of adverse reaction. Nevertheless, there is mounting evidence on the utilisation of CEUS in neonatal and premature brains, with no significant adverse events reported [40, 41]. Data from the EFSUMB paediatric CEUS registry does suggest that there may be a higher incidence of hypersensitivity or anaphylactic reactions in children compared to adults [32], and experts in the field recommend that staff performing contrast US examinations in children be properly trained and have resuscitation equipment readily available [32, 42].

### Limitations

Regulatory approval of US contrast agents in paediatric practice also continues to be challenging, and in many countries, the use of US contrast agents remains off-label for children. Ethical considerations also play a role in the controversy surrounding the utilisation of US contrast agents in children. With the US contrast agents being administered off-label in most cases, informed consent must be obtained from parents or guardians. This means explaining potential risks and benefits of a non-approved product to non-medical individuals, which may pose challenges and raise questions around the adequacy of the consent process.

### Evidence summary

**Fig. 2:**
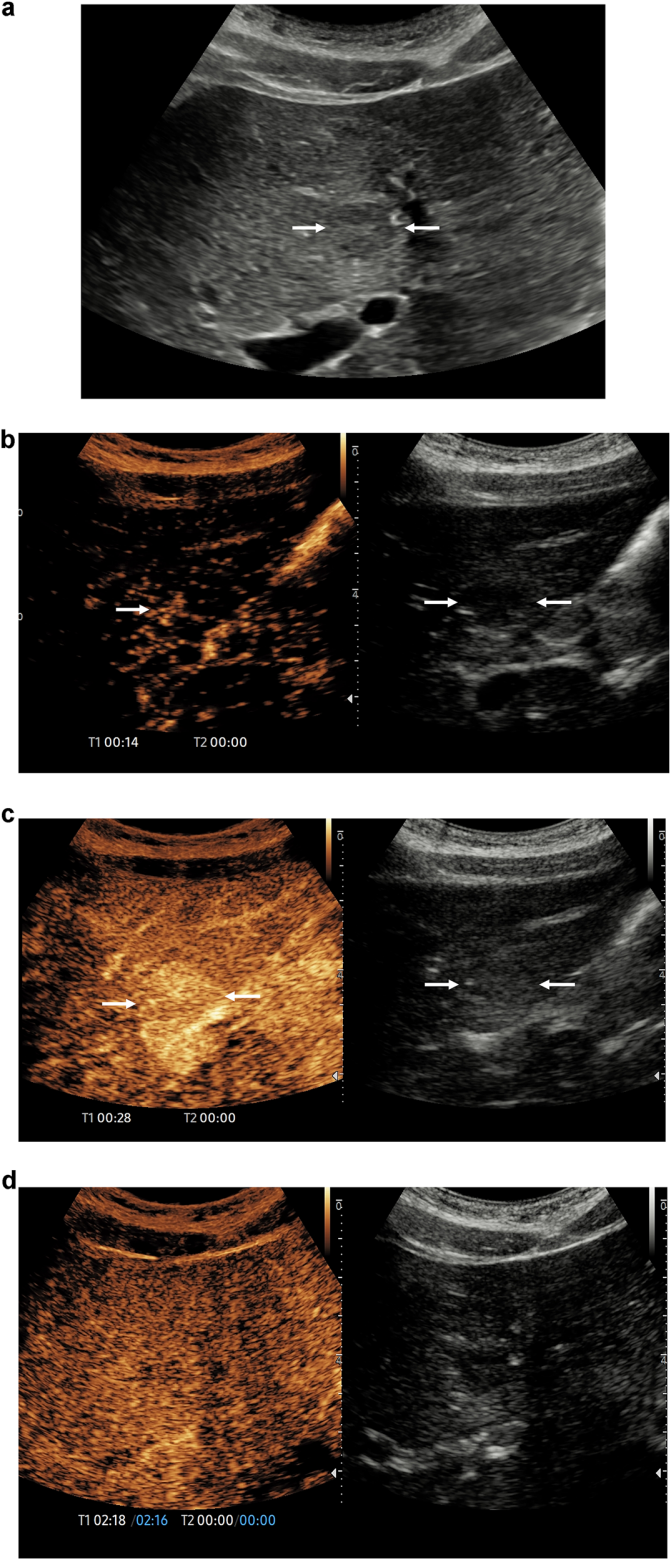
A 14-year-old female patient with chronic liver disease, a consequence of autoimmune hepatitis, found to have a new focal lesion on a liver ultrasound examination, and demonstrating indeterminate ultrasound features. **a** A focal 12 × 11 mm low reflective liver lesion(arrows) on a background of a heterogenous liver. **b** Following the administration of 2.4 mL of SonoVue™ (Bracco SpA, Milan), during the arterial phase at 14 s, there is a central spoke wheel arterial pattern (single arrow), in the focal liver lesion (arrows). This raises the possibility of the presence of an area of focal nodular hyperplasia. **c** At 28 s, during the early portal venous phase, the focal live lesion is hyper-enhancing (arrows) in comparison to the background liver parenchyma. **d** At 2 min 16 s, the late portal venous phase, there is similar washout to the background liver parenchyma, a feature of benign disease, and confirming the presence of an area of focal nodular hyperplasia

In the United States the Food and Drug Administration (FDA) approved, in 2016, the use of Lumason™ (Bracco SpA, Milan; SonoVue™ in Europe and Asia) for the characterisation of paediatric focal liver lesions [43], on the back of a series of 44 children with focal liver lesions published in 2013 by King’s College Hospital, London [44], thus encouraging sonologists across the world to use it in this context (Fig. 2). The call for acknowledgement of CEUS as a valid, advantageous technique to image children was further reinforced by EFSUMB through a position statement produced in 2016 [4], but still received criticism from paediatric radiologists and trauma surgeons [45–48]. Despite remaining off-label, the application of UCA in paediatrics has since expanded beyond focal liver lesion characterisation, with abdominal trauma [49, 50], interventional radiology [51], and oncology [52] amongst the most valuable and effective applications. CEUS has also shown promising results in the evaluation of vesicoureteral reflux (VUR) in paediatric patients [53, 54].

CEUS voiding urosonography offers a radiation-free alternative to traditional fluoroscopic voiding cystourethrography (VCUG), maintaining high diagnostic accuracy while eliminating ionising radiation exposure, which is particularly beneficial for young children requiring repeated examinations.

### Future perspectives

Ultimately, for any CEUS examination conducted in children, the radiologist or sonographer performing the examination needs to be fully trained in this advanced technique and resuscitation procedures, be satisfied with the indications and clinical utility of the examination and gain consent appropriately from the family involved. The advantages of a CEUS examination are obvious; US is a child-friendly imaging examination, a parent can be in close proximity, and the real-time repeatability allows for continuous imaging, all without the need for sedation or general anaesthesia.

## Blunt abdominal trauma (BAT)

BAT, depending on the power and direction of force, can result in a wide spectrum of injuries, ranging from minor, single-system injury to life-threatening, multi-system trauma. Patients who have sustained BAT present significant diagnostic challenges in every emergency ward. In such cases, diagnostic algorithms and guidelines from surgical and emergency medicine societies primarily recommend CT imaging and focused abdominal sonography for trauma (FAST) examinations as key imaging modalities. Although CEUS has statistically higher sensitivity (0.933 vs 0.559; two-tailed, *p* < 0.001) and specificity (0.995 vs 0.979; two-tailed, *p* < 0.001) than conventional US in cases of BAT [55], it is used in only a few, mainly paediatric, emergency medical centres.

### Advantages

The CEUS examination has all the advantages of the FAST examination, while also enabling the assessment of the integrity of solid organs and possible haemorrhage within the organ through the analysis of the internal vascularisation. In addition, it allows for a detailed assessment of all larger vessels and thus vascular injuries within the abdominal cavity. In prospective trials, visualisation and characterisation of hepatic, splenic, renal, adrenal, and pancreatic injuries in the cases of BAT correlate well with CT findings in both adults and children [56–59].

CT is the method of choice for evaluating multi-organ injuries, whereas CEUS appears to be a suitable option for assessing single-organ trauma. The widespread availability of US machines allows for repeated examinations in a short time in the emergency room and subsequent follow-up without the need for patient transport. In turn, US machine miniaturisation allows for monitoring post-traumatic changes in real time at the site of injury, during patient transport, or in warfare conditions. The assessment of post-traumatic changes using CEUS can fill the diagnostic gap in countries with underdeveloped medical infrastructure, where there is a lack of medical facilities equipped with advanced diagnostic equipment such as CT and MR [60].

### Limitations

While the evaluation of multi-organ injuries is feasible with CEUS, it requires significant expertise and speed from the examiner. Moreover, administering additional doses of US contrast may be necessary, potentially leading to a prolongation of the examination time. Some authors raise CEUS limitations in detecting certain types of abdominal injuries, including diaphragmatic rupture, bowel, mesenteric and stomach injuries, as well as bile leakage from hepatic trauma involving the bile ducts, and urinoma as a consequence of renal tract injury [58, 61, 62]. CEUS diagnostic accuracy, which can be reduced in patients with obesity or post-traumatic subcutaneous emphysema, has not been explored. Regulatory barriers also play a significant role, such as the lack of CEUS licensing for trauma applications and the absence of its inclusion in the algorithms of major trauma medical societies.

### Evidence summary

Considering the benefits of using CEUS in the assessment of BAT, the question arises why this method is so underutilised in everyday clinical practice. Over the course of 20 years (2004–2024), searching one of the largest medical databases (MEDLINE) revealed only 11 original studies involving 2040 adults—6 prospective studies (796 participants) [56, 63–66] and 5 retrospective studies (1244 participants) [67–70]. Additionally, 6 original studies involving 484 children were identified—2 prospective studies (45 participants) [58, 59] and 4 retrospective studies (439 participants) [39, 62, 71, 72].

A critical evaluation of the studies mentioned reveals several key observations. The number of adults and children studied is twice as high in the retrospective group compared to the prospective group (1683 vs 841 participants). The largest cohorts focus primarily on injuries to two organs, the liver and spleen. Studies show significant variability in their design, including differences in the time gap between CT and CEUS examinations, the number of contrast agent administrations, the scanning time allocated for each organ, the order of scanning, as well as the duration and contrast dose used. Finally, the studies do not provide unified injury scoring systems for solid organs that are directly comparable to those used in CT imaging.

### Future perspectives

**Fig. 3:**
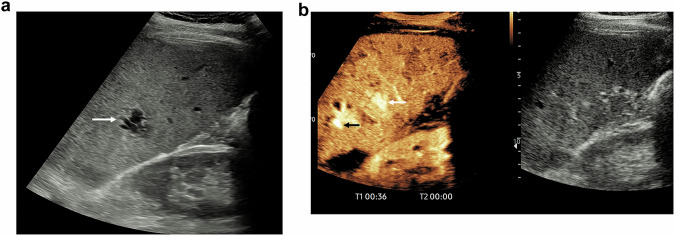
A 39-year-old male patient with BAT from a motor-bike accident, imaged by a targeted ultrasound examination to the site of small splenic laceration injury seen on the admission CT examination, day 5 post injury. **a** The B-mode ultrasound examination demonstrates a focal 12 mm low reflective irregular abnormality (arrow) without clear demarcation of a splenic laceration. **b** Following the administration of 1.2 mL of SonoVue™ (Bracco SpA, Milan), during the late arterial phase at 36 s, two pseudoaneurysms are present, the larger (white arrow) was seen on the B-mode examination, whilst the other (black arrow) was not seen. Further follow-up ultrasound examinations demonstrated resolution of the pseudoaneurysms

On the other hand, CEUS has proven to be a valuable and radiation-free tool for the follow-up of paediatric patients with post-traumatic splenic, hepatic, and renal injuries (Fig. 3). It demonstrates high sensitivity and specificity in detecting complications such as pseudoaneurysms, making it a reliable alternative to CT for pseudoaneurysm monitoring [49, 73]. To enhance its adoption, future research should focus on standardising CEUS protocols, developing unified injury scoring systems, and addressing diagnostic limitations in complex injuries. Additionally, overcoming regulatory barriers and integrating CEUS into trauma guidelines could expand its role in clinical practice, particularly in resource-limited settings and paediatric populations.

## Complex renal cysts

Renal cysts are common, especially in the elderly, with a prevalence at Imaging in a range of 4–41%, depending on the patient spectrum and the sensitivity of the imaging modality [74]. Cystic lesions are classified as non-neoplastic, benign and malignant; most are benign, but in the setting of chronic renal disease have a higher risk of malignant degeneration. There are several guidelines for the diagnosis and management of renal cystic lesions [75–77].

The Bosniak classification (I-IV, Table 1)), a risk stratification tool for the characterisation of cystic renal lesions based on contrast-enhanced computed tomography (CECT), has been the gold standard for almost 30 years [78], established to improve the management of atypical cystic lesions to avoid unnecessary surgery [79]. However, limitations of the CECT-based classical Bosniak classification are notable: repetitive radiation exposure for follow-up of benign lesions, not including benign neoplasia and reflecting the technological developments of the past three decades, use of the least appropriate imaging modality for the evaluation of cystic lesions in general. Biopsy of atypical cystic masses is not part of the routine management due to its limited accuracy in Bosniak IIF and III lesions, the theoretical risk of dissemination and the changes in imaging features that will make follow-up difficult.

**Table 1:** CEUS of renal cysts according to the Bosniak classification, EFSUMB 2020 proposal for a CEUS-adapted Bosniak cyst categorisation [5]

❖ Category l:
• A simple benign cyst shows on ultrasound, CT and MR imaging a sharp interface with adjacent renal parenchyma, no wall thickening. Ultrasound is typically echo-free with acoustic posterior enhancement.
❖ Category II:
• These lesions are minimally complicated cysts, that can be easily classified as benign. Single thin (≤ 1 mm) septa and fine calcification in their walls or in the septa can be found. Diameter < 3 cm.
❖ Category IIF:
• These lesions are more complicated but cannot be confidently classified as Bosniak III. Several thin (≤ 1 mm) septa. 3 cm or more in diameter.
❖ Category III:
• These lesions cannot be definitely characterised as benign or malignant. They show some signs of malignancy and should be considered as potentially malignant. Such signs are irregular walls, thick calcification, or multiple thickened and enhancing septa.
❖ Category IV:
• Their cystic lesions show definite criteria of malignancy, like thickened, irregular and enhancing parts of the tumour. Among these are also tumours with contiguous cystic parts or malignant tumours that occur within the wall of a cyst.

### Evidence summary

An update in 2019 of the Bosniak classification was proposed by Silvermann et al [76] to reduce the interobserver variability, particularly among readers with variable experience, providing a precise description of the cystic walls and septations, as well as the specificity of malignancy, particularly for category IIF and III lesions. A recent meta-analysis demonstrated significant improvements in specificity (0.62 vs 0.41, *p* < 0.001) in risk stratification of complex cystic renal lesions at the cost of a moderate but significant decrease in sensitivity (0.88 vs 0.94, *p* = 0.001). In radiological imaging, irregular septa, thickness of more than 2 mm, nodular changes, irregular wall thickening, and significant contrast enhancement indicate malignancy [80, 81]. Calcification is a non-specific sign [82]. In CECT, the presence of nodular or septal enhancement showed the highest sensitivity for predicting malignancy with moderate to good interobserver agreement. All cystic renal cell carcinomas had an enhanced septal or nodular component. The mean sensitivity and specificity in predicting malignancy for the presence of septal enhancement were 83 and 82%, respectively; for nodular enhancement, 67% and 96%; and for septal or nodular enhancement, 100% and 86%, respectively [83].

In a CEUS study [84] with histopathologically confirmed Bosniak III cysts, 62% had septa, of which 61% were malignant. Again, 75% of all focal thickened and contrast-enhanced septa were malignant [85]. According to the 2019 Bosniak classification, cystic masses with many or thick enhancing septa are associated with a higher likelihood of malignancy than masses with few or thin septa. The total volume of enhancing tissue in a cystic mass may be the most important indicator of whether that mass is benign or malignant [76].

While in past it was postulated that CECT should be the referral imaging modality to characterise renal cysts, it is not cost-effective. Furthermore, potentially benign lesions may be monitored by CT or MR imaging, with the risk related to radiation exposure. It has been suggested that CEUS is an ideal Imaging modality in this setting [5]. In a recent systematic review and meta-analysis of retrospective studies, the application of CECT, contrast-enhanced MR imaging, and CEUS in the evaluation of renal cystic lesions was investigated, with ten relevant articles identified [85]. The meta-analysis indicated that CEUS had high sensitivity and specificity in diagnosing renal cystic lesions, which was statistically significant. Even more recently, Munch et al [86], showed that categorisation of cystic renal lesions based on the Bosniak classification proposed by EFSUMB in 2020 showed very good reproducibility. While even less experienced observers achieved mostly substantial agreement, training remains a major factor for better diagnostic performance.

### Advantages

CEUS does not use ionising radiation, is not nephrotoxic (renal function tests are not required), is inexpensive, preferred by patients with vascularisation evaluation by both colour Doppler US and CEUS examination, and US contrast agents are safer than both iodinated contrast agents and gadolinium-based contrast agents. CEUS examination performs better than CECT in the detection of lesion vascularity, depicts more septa and is superior in depicting the degree of both septae and wall thickening, septal enhancement and enhancement of solid component within the lesion. CEUS is extremely sensitive in revealing even the tiny capillaries that feed hairline-thin septa with a superior temporal and spatial resolution. However, it is operator experience dependant, it can be limited by challenging patient habitus, bowel gas or ribs, and by the patient’s compliance. The examiner must be appropriately qualified in CEUS, with structured report templates helpful in ensuring quality [87].

### Limitations

Contraindications to the use of CEUS, inadequate visualisation of the target area and the lack of expertise are the main limitations.

### Future perspectives

When US visualisation is adequate and local expertise is available, immediate characterisation of incidentally found features and effective follow-up may be accomplished. CEUS permits characterisation of indeterminate lesions at CT or MR imaging, can be used in patients who cannot tolerate CT and MR imaging or when kidney insufficiency is present; CEUS is the most cost-effective technique [88]. The role of CECT in complex renal cyst evaluation and follow-up should diminish.

**Fig. 4:**
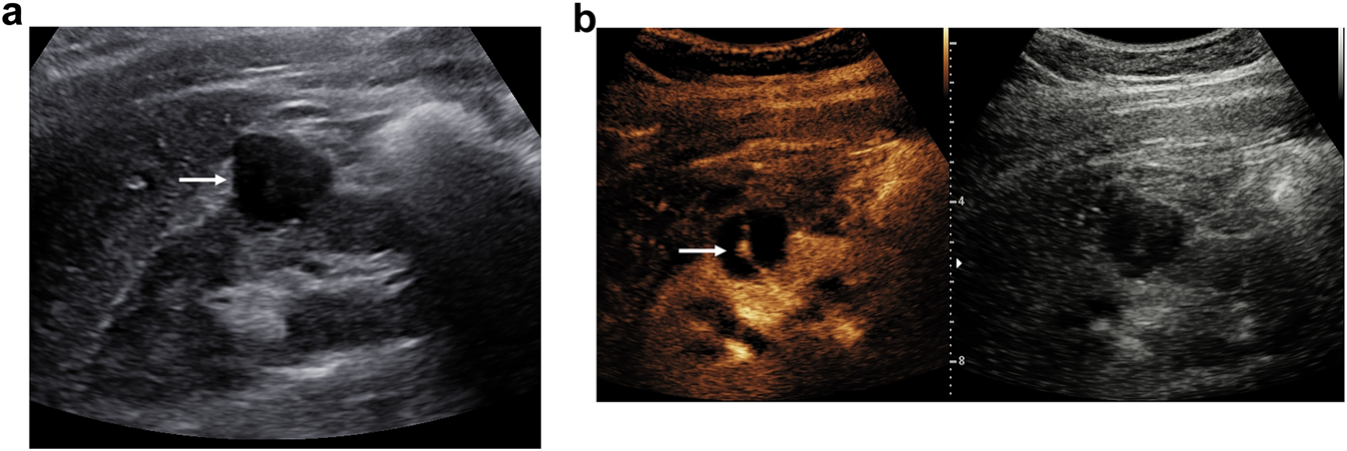
A 78-year-old male patient with ‘acute kidney injury’, undergoing a routine examination of the kidneys. **a** On the B-mode ultrasound examination, there is an 18-mm cystic structure at the mid aspect of the right kidney, with some debris within. **b** An immediate CEUS examination, which is safe in the presence of renal impairment, using 1.2 mL of SonoVue™ (Bracco SpA, Milan), there is enhancement of a > 2-mm septation (arrow) within the cyst, classifying this a Bosniak type IIF

This raises important questions for the radiologist. Why should predominantly benign cysts be repeatedly examined by CECT over a period of years, using a radiation-intensive procedure, while this is also possible using a less expensive procedure with fewer side effects? MR imaging can be used for Bosniak III cysts and is recommended in Bosniak IV before surgery. CT imaging is better applied for staging malignancies prior to surgery (Fig. 4).

## Endovascular aortic stent grafts

Endovascular aneurysm repair (EVAR) is a minimally invasive procedure used to treat abdominal aortic aneurysms (AAA) by placing a stent-graft within the aorta to exclude the aneurysm, thereby reducing the risk of rupture [89]. This has become a commonly performed procedure to treat AAA, with much reduction in patient morbidity, and is favoured by clinicians and patients [90]. Post-EVAR surveillance is crucial to monitor for complications such as endoleaks, graft migration, and aneurysm sac enlargement [91]. Traditionally, CECT has been the standard imaging modality for post-EVAR follow-up, with surveillance examinations performed for life [92]. Graft migration can be assessed using plain X-rays, with endoleaks needing cross-sectional imaging for classification and management; Endoleaks are classified Types I–V, with Type II endoleaks the most common [93]. The application of CEUS for the monitoring of endoleaks is a valuable imaging technique offering several advantages in monitoring stent-graft status [94, 95].

### Advantages

One of the primary benefits of CEUS is the absence of ionising radiation, making it a safer option for patients requiring lifelong surveillance, although the majority of patients undergoing an EVAR procedure are elderly, a basic principle has to be observed to reduce radiation exposure in all patients [96, 97]. The lack of nephrotoxicity with the use of CEUS is likely of more importance in the elderly EVAR patient, often with renal impairment associated with a generalised vascular atheromatous condition, where repeated iodinated contrast examinations of the CECT examination will compromise renal function [98]. There is also the added advantage of lower cost and real-time evaluation [99, 100].

### Evidence summary

CEUS has shown high diagnostic accuracy in detecting endoleaks, particularly type II endoleaks, which are the most common and involve retrograde flow from collateral vessels, with follow-up examinations being accurate [101]. In a study comparing CEUS and CECT, CEUS demonstrated similar sensitivity and specificity in detecting endoleaks, suggesting it can be effectively integrated into EVAR surveillance protocols [102]. With Type V complications, so termed ‘endotension’, CEUS accurately detected more leaks than CECT [103, 104].

### Limitations

**Fig. 5:**
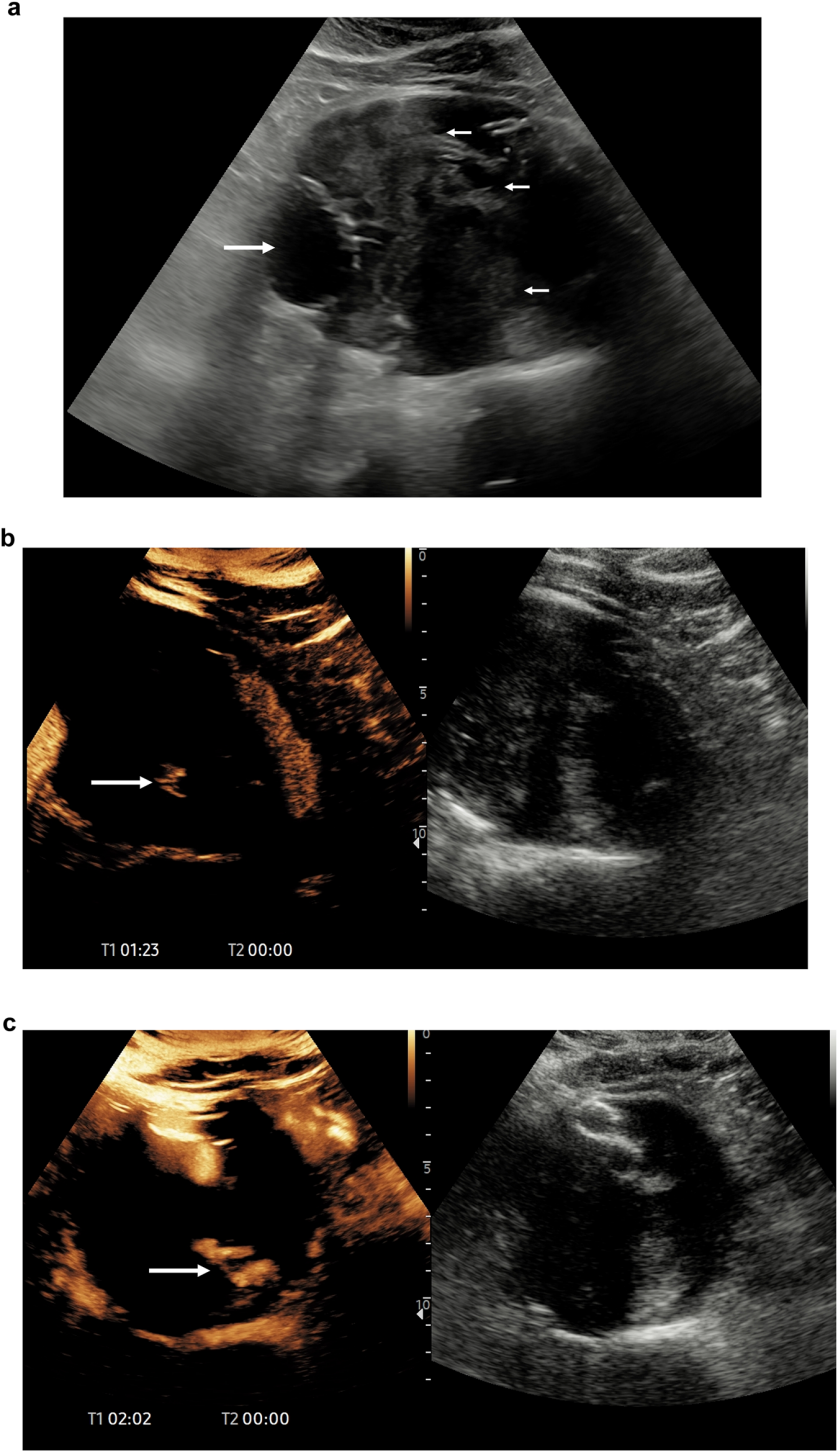
An 81-year-old man underwent an EVAR three years prior, with repeated CT examinations showing a persistent sac dilatation and no endoleaks. The patient was referred for a CEUS examination. **a** The baseline B-mode ultrasound examination demonstrates the aortic stent graft (arrow) with mixed echogenic contents of the aortic sac (small arrows), suggesting evolving haematoma. **b** Following the administration of 1.2 mL of SonoVue™ (Bracco SpA, Milan), at 1 min 23 s post-administration, there is an endoleak demonstrated (arrow). **c** At 2 min 2 s, on a maximum intensity projection, there is accumulation of contrast (arrow) at the site of the endoleaks from posterior lumbar vessels. The CECT examinations did not show the late endoleaks, whereas the real-time continuous examination with ultrasound enabled a longer examination to demonstrate the endoleak

Despite many advantages, CEUS has limitations. Its effectiveness can be reduced in patients with high body mass index or excessive bowel gas, which may compromise image quality. Additionally, CEUS is operator-dependent, requiring specialised training and experience to accurately interpret findings [105]. In some cases, CEUS may not visualise the entire endograft, particularly in deeper anatomical regions, and recognition of artefacts is important [106] (Fig. 5).

### Future perspectives

The ability to add fusion imaging to the CEUS examination, where the dynamic CEUS examination is fused with the most recent CT, allows precise anatomical location of any leak and allows treatment options [91, 107]. Adequate training for the practice of CEUS analysis of aortic stent graft follow-up is paramount to increasing the availability of this patient-friendly technique.

## Summary (Table 2)

### Pregnancy

There is limited data on the use of US contrast agents in pregnancy, but the agents used are truly intravascular and do not cross the placental barrier. Clinical reports using CEUS in pregnancy demonstrate the lack of any contrast enhancement in the foetus during the US examination, confirming the absence of macrovascular microbubbles in the foetus.

**Table 2:** Summary of evidence for CEUS in different clinical scenarios

Clinical scenario	Sensitivity	Specificity	Advantages	Limitations
Pregnancy	Limited data; case reports show diagnostic utility [10, 26, 27]	Limited data; case reports confirm no foetal contrast enhancement [10, 26]	- Non-ionising, avoids radiation and iodinated contrast - Safe intravascular microbubbles do not cross placental barrier [15–17] - Suitable for maternal conditions (e.g. hepatic lesions and abscesses) [10, 26, 27]	- Lack of formal approval by EFSUMB, WFUMB, ISUOG [13] - Sparse prospective data; mainly case reports [22–24, 26, 27] - Potential unknown foetal effects require further research
Paediatric practice	High for focal liver lesions (FDA-approved) [43, 44]; limited data for other applications [32, 38, 39]	High for focal liver lesions [43, 44]; limited data for other applications [32]	- Radiation-free, child-friendly, and no sedation needed - Allows parental presence during examination - Effective for liver lesions, trauma, and oncology [4, 49, 50, 52]	- Off-label use in most countries (except US for liver) [4, 43] - Limited paediatric safety data; potential higher hypersensitivity risk [32] - Requires trained operators and resuscitation readiness [32, 42]
BAT	0.933 (vs 0.559 for conventional US, *p* < 0.001) [50]	0.995 (vs 0.979 for conventional US, *p* < 0.001) [50]	- High sensitivity/specificity for solid organ injuries [50] - Radiation-free, repeatable, portable for emergency settings [58] - Effective for paediatric follow-up (e.g. pseudoaneurysms) [49, 71]	- Limited for multi-organ or specific injuries (e.g. diaphragmatic, bowel) [56, 67, 70] - Operator-dependent; requires expertise and speed [58] - Regulatory barriers; not in major trauma algorithms [50]
Complex renal cysts	High (meta-analysis; exact values not specified) [84]	High (meta-analysis; exact values not specified) [84]; 86% for septal/nodular enhancement [81]	- Superior to CECT for detecting septa, vascularity [5, 84] - Non-ionising, non-nephrotoxic, cost-effective [86] - Good reproducibility with EFSUMB 2020 Bosniak classification [84]	- Operator-dependent; limited by patient habitus, bowel gas [85] - Less effective for staging malignancy (CT preferred) [86] - Requires structured reporting and training [84, 85]
Endovascular aortic repair (EVAR) follow-up	Similar to CECT for endoleaks (exact values not specified) [100]; higher for Type V endotension [101, 102]	Similar to CECT for endoleaks (exact values not specified) [100]	- Radiation-free, non-nephrotoxic, and ideal for lifelong surveillance [94, 96] - High accuracy for Type II endoleaks [99] - Cost-effective, real-time imaging [98, 99]	- Limited by high BMI, bowel gas, or deep anatomy [105] - Operator-dependent; requires specialised training [105] - May not visualise entire endograft [106]

Sensitivity and specificity are directly cited where available (BAT [50], and complex renal cysts [84]). For scenarios with limited quantitative data (pregnancy, paediatric practice), qualitative evidence is summarised from case reports or registries [10, 26, 27, 32, 43]

### Paediatric

The use of US in paediatric practice is vital, and the addition of a US contrast agent would seem logical for the well-being of the child. The ability to perform a CEUS examination that prevents CT or MR imaging is beneficial, with the presence of the parent during scanning ideal. There seems to be a slightly higher allergic reaction rate than in adults, but it is still very low, with a paucity of safety data.

### Trauma

CEUS offers significant advantages in the assessment of BAT, particularly its ability to evaluate vascularisation and detect bleeding in solid organs without exposing patients to ionising radiation, particularly beneficial for vulnerable populations such as children and pregnant women. Despite these benefits, CEUS remains underutilised due to limitations in detecting multi-organ/certain types of injuries, the need for high operator skill and regulatory barriers, including its restricted licensing for paediatric applications and lack of integration with major trauma algorithms.

### Complex renal cysts

CEUS is a non-invasive and cost-effective modality for the classification of renal Bosniak cysts, due to the inherent excellent resolution of US, the smallest vascularised structures can be made visible, for the management of typical and atypical cystic masses, limiting MR imaging to specific situations. Vascularity is the hallmark of malignancy. Limitations are patient-dependent body habitus, with CT often not needed except for staging in malignancy.

### Endovascular aortic repair with stent grafts

CEUS has emerged as a valuable tool in the surveillance of patients post-EVAR, offering a radiation-free and nephrotoxic-free alternative to CECT. Its high diagnostic accuracy in detecting endoleaks, particularly type II, makes it a reliable option for routine follow-up. However, patient selection and operator expertise are crucial to optimise its effectiveness, requiring further studies and advancements in US technology to enhance the applicability and accuracy of CEUS in EVAR surveillance. CEUS has the potential as a first-line imaging modality in this setting.

## Conclusion

Although the discussion revolves around the controversies using microbubble contrast agents outside the limited licensed uses, this limitation is not borne of practicality and logical medical imaging practice. The agent commonly used in Europe, SonoVue™, is truly intravascular with no extra-cellular phase, not nephrotoxic, is metabolised rapidly, with the phospholipid shell broken down in the liver and the inert sulphur hexafluoride gas exhaled via the lungs. The agent does not cross the placental barrier. The agent is present in the entire vascular tree, and the vessels are imaged at a point in time ‘through’ the overlying organ; a whole body license is appropriate [8], as is allowed with the use of other imaging contrast agents and iodinated and gadolinium agents being less safe than US contrast agents. The non-hepatic guidelines of EFSUMB [2, 3], a well-respected scientific society, have indicated that there is enough evidence for the safe application of ultrasound contrast agents outside licensed uses, with support of regulatory authorities [1]. There should be widespread application of CEUS imaging in these areas without delay for the betterment of patient care.

## Abbreviations

BAT: Blunt abdominal trauma
CEUS: Contrast-enhanced ultrasound
CT: Computed tomography
EFSUMB: European Federation of Societies in Medicine and Biology
EVAR: Endovascular aneurysm repair
MR: Magnetic resonance
US: Ultrasound
